# Renal Apparent Diffusion Coefficient Values in Patients with Obstructive Uropathy and High Values of Creatinine

**DOI:** 10.5334/jbr-btr.854

**Published:** 2015-09-15

**Authors:** F. Sonmezgoz, T. O. Kalaycı, A. Karakeci, Ş. Karasu

**Affiliations:** 1 Firat University, Faculty of Medicine, Department of Radiology, Elazig, Turkey; 2 Izmir Ataturk Training and Research Hospital, Department of Radiology, Izmir, Turkey; 3 Firat University, Faculty of Medicine, Department of Urology, Elazig, Turkey

**Keywords:** Kidney, stenosis or obstruction – Kidney, MR

## Abstract

**Purpose:** Diffusion weighted magnetic resonance imaging (DW-MRI) of the kidneys provides noninvasive information on renal function in healthy volunteers, and it is feasible in severely ill patients. It may provide information on the degree of kidney dysfunction.

The purpose of this study is to evaluate apparent diffusion coefficient (ADC) values using DW-MRI in patients with obstructive uropathy and elevated serum creatinine levels.

**Methods:** Fifty patients with high serum creatinine levels and diagnoses of chronic urinary obstruction detected by ultrasonography were included in this study.

DW-MRIs were obtained from 50 patients with hydronephrotic kidneys and 26 healthy volunteers using a 1.5T whole-body MR scanner.

**Results:** ADC measurements of renal parenchyma in hydronephrotic kidneys were significantly lower compared to normal kidneys (p < 001).

**Conclusion:** The measurement of ADC values has potential value in the evaluation of the functional status of hydronephrotic kidneys.

Urinary tract obstruction that causes hydronephrosis may lead to an increased pressure proximal to the obstruction, including the tubules and the renal interstitium, thus causing tubular dysfunction, a decrease in glomerular filtration rate (GFR) and increased serum creatinine levels. Early diagnosis and treatment of obstructive hydronephrosis is important to preserve renal function. Imaging studies have a key role in diagnosis and follow-up of the renal obstruction [1]. Detailed morphological information including kidney size can be obtained from ultrasonography imaging (US). To a certain extent, renal blood-flow measurements, and intrarenal resistive index can be used to estimate the degree of obstruction. Yet, the degree of dilatation is not related to the degree of obstruction, and resistive index may be completely normal in chronic obstruction, or may be abnormal due to other renal parenchymal disease processes. Computed tomography (CT) is a method that can provide information on renal morphology, and on the site and cause of obstruction [2]. Individual renal function is usually evaluated by nuclear medicine techniques, which are considered as more reliable. However, these techniques have some disadvantages, such a low spatial resolution. GFR and effective renal plasma flow can be evaluated by this technique, but renal diffusion and perfusion cannot be assessed. All techniques suffer from similar disadvantages, i.e. not providing quantitative information. Diffusion weighted magnetic resonance imaging (DW-MRI) of the kidneys gives non-invasive information on renal function in healthy volunteers, and it is feasible in severely ill patients. Quantitative data on the degree of renal dysfunction may be obtained [3].

The purpose of this study is to evaluate the value of apparent diffusion coefficient (ADC) values from DW-MRI in patients with obstructive uropathy and elevated serum creatinine levels.

## Materials and methods

The study protocol was approved by the local ethics committee, and informed consent was obtained from all volunteers and patients.

### Study population

Fifty patients (40 men and 10 women; mean age, 61 ± 16.9 years; age range, 24–78 years) with increased serum creatinine levels (2 ± 0.89) mg/dL and the diagnosis of chronic urinary obstruction due to urinary stone, tumour or fibrosis were included in this study. All patients had a history of obstructive uropathy longer than 6 weeks. The control group consisted of 26 healthy volunteers (8 men and 18 women; mean age, 49 ± 18.8 years; age range, 21–95 years) who had no history of renal disease and had normal creatinine levels (0.7 ± 0.12). All volunteers were referred for upper abdominal magnetic resonance imaging (MRI) for hepatic haemangioma. Serum creatinine values were obtained from all patients on the day of the MRI examination.

### MR Imaging

MRI examinations were performed on a 1.5T whole-body superconducting MR scanner (General Electric Signa HiSpeed scanner, Milwaukee, WI, USA) with high-speed gradients. A body coil was used for all sequences. Axial T2-weighted fat saturation spin-echo images (TE: 90, TR: 5700, slice thickness: 8 mm, intersection gap: 1.5, number of excitation: 4, matrix size: 512 × 512) were obtained from each patient for demonstration of the excretory system. Diffusion weighted images (TE: 72, TR: 8000, FOV: 30 × 30, slice thickness: 5 mm, intersection gap: 0, number of excitation: 1, matrix size: 128 × 128) were obtained using single-shot spin-echo, echo-planar imaging (EPI) sequences with diffusion gradient *b* values of 100, 600 and 1000 s/mm^2^. Maximum 26–40 number of slices was obtained, examination time was 30 sec and direction of diffusion all. All images were obtained without breath holding.

### Image analysis

**Figure 1:**
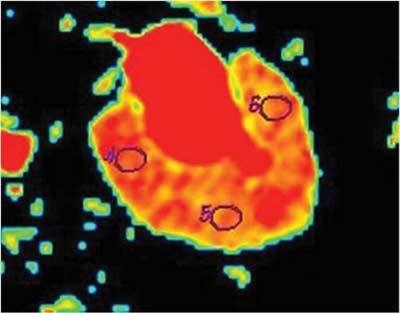
Axial ADC map calculated from echo-planar DWI of hydronephrotic kidneys with high *b* value; the ROIs are placed in the middle portion of the kidney at three locations: anterior lip, intermediate site and posterior lip.

The DW-MRI data were transferred to a workstation (Advantage Windows, software version 2.0, General Electric Medical Systems, Milwaukee, WI, USA). Monoexponential fitting of the diffusion decay curves were utilized. A circular region of interests (ROI) was placed at the corticomedullary junction for the measurement of ADC values in normal and obstructed kidneys. For each kidney, three ROIs were placed in the middle portion (Fig. 1). For each ROI, the mean ADC-values and standard deviations were calculated. All measurements were repeated using different *b* values (100, 600 and1000 s/mm^2^). The ADC maps were calculated automatically with the MR-system, and ADC values were expressed in square millimetres per second (mm²/s).

### Statistical analyses

Statistical analyses were performed with the SPSS 12.0 software package program. The ADC values of the volunteers and patients were reported as the mean ± standard deviation. Independent samples *t* test was used for the comparison of parenchymal ADC values and the comparison of the values of normal kidneys and obstructed kidneys. A *p* value of less than .05 was considered a statistically significant.

## Results

DW-MRI studies were of diagnostic quality in all cases, and no cases were excluded from this study. Seven of the patients had bilateral hydronephrosis. DW-MRI of 57 hydronephrotic kidneys were obtained from 50 patients, and images of 52 presumably healthy kidneys were collected from 26 volunteers.

**Figure 2:**
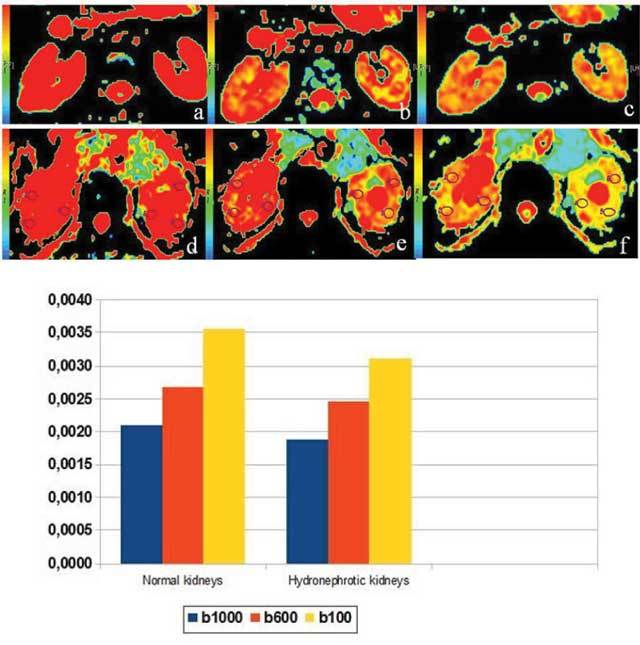
Axial ADC maps of normal non-obstructed (a–c) and hydronephrotic kidneys (d–f) with *b100* (a, d), *b600* (b, e) *and b1000* (c, f) values; the red colour represents significantly high ADC values and whereas the yellow-green colour displays lower ADC values.

With increasing *b* values, a significant decline in renal signals was observed in the normal and hydronephrotic kidneys. ADC maps were created from diffusion weighted echo-planar images and displayed in colour. The colour map was dependent on increasing b values and decreasing ADC values in both normal and hydronephrotic kidneys (Fig. 2).

**Figure 3:**
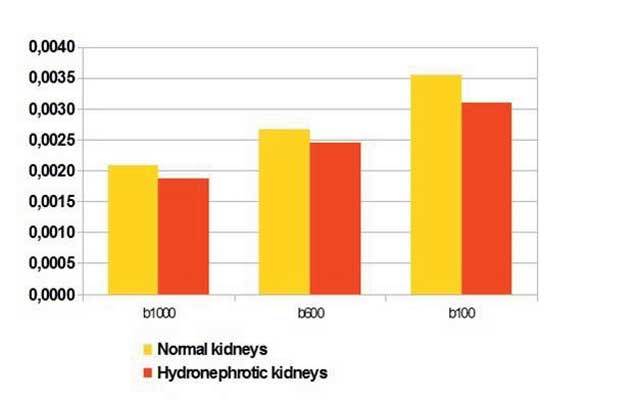
Comparison between mean ADC values of obstructed kidneys and normal kidneys for *b1000, b600* and *b100* (mm^2^/s).

The mean ADC values of normal kidneys for b100, b600 and b1000 values were (3.55 ± 0.29) × 10^–3^, (2.67 ± 0.49) × 10^–3^ and (2.09 ± 0.19) × 10^–3^ mm^2^/s, respectively. The mean ADC values of obstructed kidneys with b100, b600 and b1000 values were (3.10 ± 0.56) × 10^–3^, (2.45 ± 0.55) × 10^–3^ and (1.87 ± 0.26) × 10^–3^ mm^2^/s, respectively. Mean ADC values of hydronephrotic kidneys for b100, b600 and b1000 were significantly lower than normal kidneys (p < 0.001) (Fig. 3) (Table 1).

**Table 1:** Apparent diffusion coefficient values in parenchyma of normal kidneys and obstructed kidneys for *b100, b600* and *b1000* values. * The differences are statistically significant between the groups (p < 0.001).

	*Mean ± SD*	*Mean ± SD*	*Mean ± SD*
	*b100*	*b600*	*b1000*

**Normal kidneys**	3.55 ± 0.29 × 10^–3^	2.67 ± 0.49 × 10^–3^	2.09 ± 0.19 × 10^–3^
**Obstructed kidneys**	3.10 ± 0.56 × 10^–3^*	2.45 ± 0.55 × 10^–3^*	1.87 ± 0.26 × 10^–3^*

## Discussion

The DW-MR is a technique based on the principle of measuring diffusion activity, which is the Brownian motion of the spins in biologic tissues. This technique enables the quantitative measurement of the ADC values of lesions. ADC combines the effects of capillary perfusion and water diffusion in the extracellular extravascular space. Thus, DW-MR provides simultaneous information about perfusion and diffusion in any tissue. When only high *b* values are applied, the ADC values predominately represent true diffusion. Low *b* values are influenced by both perfusion and diffusion [3]. DW-MRI has been widely used in neuroimaging for the evaluation of acute cerebral stroke, intracranial tumours and demyelinating disease processes. Until a few years ago, DW-MRI was only used in neuroimaging; application of abdominal MR-DWI was limited due to its susceptibility to respiratory motion, cardiac movement and bowel peristalsis. Recently, an ultra-fast EPI technique became available, enabling DWI of the abdominal and even of thoracic masses. Acquisition time of EPI sequences is fast (17 sec) minimizing the effects of gross physiologic motion. In the kidney structure and function are tightly linked. is the kidney is highly vascularized with a rich blood supply, the parenchyma has a high water content, and its function, very simplified, largely depends on the flow of water [4].

Several investigations have reported on ADC measurements in the human kidney. Muller et al [5] found a renal ADC value of 3.54 ± 0.47 × 10^–3^ mm^2^/s. In a second study, the researchers [6] investigated renal diffusion in subjects who were initially dehydrated and subsequently hydrated. The mean ADC value for dehydrated subjects was 2.88 ± 0.65 × 10^–3^ mm^2^/s; for hydrated subjects the mean was 3.56 ± 0.32 × 10^–3^ mm^2^/s. Fukuda et al [7] and Toyoshima et al [8] reported lower ADC values in normal kidneys (1.68 × 10^–3^ and 1.50 × 10^–3^ mm^2^/s than the ADC values that we found in our study. A large range of ADC values in human kidneys was reported in the literature. In several studies, authors evaluated ADC values separately in the cortex and in the medulla. Cova et al [9] placed the ROI cursors at the level of the corticomedullary junction anticipating that is might be difficult and inaccurate to position the ROI cursor separately on the renal cortex and medulla, consistent with Fukuda et al [7]. The researchers suggested to measure ADC values in the midportion of the kidney, which is less influenced by the perfusion effect [7]. In the present study, large ROI cursors were placed at the level of the corticomedullary junction.

Namimoto et al [10] found that the ADC values in the kidneys with acute renal failure, chronic renal failure and renal artery stenosis (RAS) were significantly lower than normal kidneys. In another study by Yildirim et al [11], ADC values in RAS kidneys were significantly lower than in normal kidneys. Chan et al [12] evaluated the DW-MRI for differentiating between hydronephrosis and pyonephrosis. They measured the ADC values from the dilated pelvicalyceal system (not the parenchyma) and found that ADC values were significantly lower in pyonephrotic kidneys.

Unilateral ureteral occlusion was performed by Muller et al [6] in pig kidneys. The ADC values of the renal cortex and medulla decreased in the obstructed kidneys. In a similar study, Pedersen et al [13] found significantly reduced ADC values in acute ureter occlusion and increased renal parenchymal ADC values in chronic unilateral partial obstruction.

Thoeney et al [3] evaluated the feasibility of DW-MRI of renal function in healthy volunteers and patients with various renal abnormalities. They did not find substantial differences in the average ADC values compared to the contralateral kidneys. In a similar study, Bozgeyik et al [14] found statistically insignificant lower ADC values in acute obstructed kidneys compared to normal kidneys.

Mechanical obstruction of the urinary outflow rises the luminal pressure and with dilatation of the proximal collecting system. Rise in luminal pressure increases the interstitial pressure in the cortex and medulla and decreases renal blood flow and thus contributes to decrease in ADC values [3]. In the present study, significantly lower ADC values were found in hydronephrotic kidneys compared to healthy kidneys. These findings may be related to the duration of the obstruction in the cases included in the study. Hence, the degree and duration of obstruction are key factors in renal DW-MRI.

With new developments, probably the role of functional MRI will increase in the management of patients with renal disease. These techniques include perfusion studies for assessment of the renal blood flow and measuring the renal function with blood-oxygen level-dependent imaging techniques or dynamic contrast-enhanced studies. Contrast-enhanced dynamic MRI can measure the time-dependent concentrations of Gd-DTPA in the cortex and the medulla that reflect renal function [15].

Currently, the gold standard examinations are normally performed for measuring kidney perfusion and function, namely the radioisotopic methods which can provide reliable results with excellent reproducibility concerning glomerular filtration rate, effective renal plasma flow, renal perfusion and urine drainage. The main limitation of our study is, there is no gold standard correlation such as kidney radioisotope examination.

## Conclusion

DW-MRI is a non-invasive method that gives reproducible data reflecting renal function. The measurement of ADC values by DW-MRI has potential value in the evaluation of the functional status of hydronephrotic kidneys. The ADC values of renal parenchyma in obstructive uropathy are significantly lower than in normal kidneys. However, further investigations are needed to support these results and to evaluate the usefulness in clinical practice.

## Competing Interests

The authors declare that they have no competing interests.

## Note

This study was presented as an e-poster at the *European Congress of Radiology (ECR)* Vienna, Austria, March 6–10, 2014.
